# Persistent pulmonary hypertension of the newborn due to methylmalonic acidemia: a case report and review of the literature

**DOI:** 10.1186/s13256-023-04031-8

**Published:** 2023-07-11

**Authors:** Fariba Hemmati, Hamide Barzegar

**Affiliations:** Neonatal Research Center, Shiraz University of Medical Sciences, Shiraz, Iran

**Keywords:** Methylmalonic acidemia, Persistent pulmonary hypertension of the newborn, Inborn errors of metabolism, Organic acidemias, Metabolic acidosis, Persistent fetal circulation, Newborn, Case report

## Abstract

**Background:**

Persistent pulmonary hypertension of the newborn manifesting with refractory and severe cyanosis is the consequence of high pulmonary vascular resistance causing extrapulmonary right-to-left shunt. Acidosis and hypoxemia produce pulmonary vasoconstriction. Persistent pulmonary hypertension of the newborn occurs due to numerous disorders and has been rarely reported as a manifestation of methylmalonic acidemia. We report a newborn with methylmalonic acidemia who presented with persistent pulmonary hypertension of the newborn.

**Case presentation:**

A 1-day-old Iranian girl presented with respiratory distress and refractory metabolic acidosis. She was born at 39 + 5 weeks gestational age with Apgar scores of 8 and 9 in the 1st and 5th minutes, respectively, and was in good condition up to 10 hours of life. After that, she presented with cyanosis, tachypnea, retraction, and hypotonia. Despite receiving oxygen, she had low oxygen saturation. Echocardiography revealed severe pulmonary hypertension and right-to-left shunt through patent ductus arteriosus and foramen ovale. Her acidosis worsened despite receiving full support and medical therapy. So, she was started on peritoneal dialysis. Unfortunately, she did not respond to treatment, and after she had died, biochemical tests confirmed methylmalonic acidemia.

**Conclusion:**

Persistent pulmonary hypertension of the newborn is a very rare manifestation of methylmalonic acidemia. Severe inborn errors of metabolism may cause irreversible damage with adverse lifelong morbidity, and early diagnosis may help to prevent such complications. Furthermore, diagnosis of these disorders aids in prenatal diagnosis through the use of cultured amniocytes or chorionic villi to detect gene mutations, as well as biochemical analyses of amniotic fluid for subsequent pregnancies.

## Background

Methylmalonic acidemia (MMA), the most common form of organic acidemia [1], is the result of methylmalonyl COA mutase (MCM) enzyme deficiency or defect in synthesis or transport of its coenzyme, adenosylcobalamin (AdoCbl), or deficiency of methylmalonyl-CoA epimerase enzyme [2, 3]. Acute clinical presentation of MMA is sepsis-like, and nonspecific symptoms such as vomiting, poor feeding, lethargy, respiratory distress, decreased level of consciousness, hypo-/hypertonia, seizure, and temperature instability may develop [4]. Cardiomyopathy, arrhythmias and sudden death were also reported without clear pathogenesis [5, 6]. Laboratory findings include severe and persistent metabolic acidosis, hypoglycemia or hyperglycemia, ketonuria, hyperammonemia, leukopenia, and thrombocytopenia [3].

Persistent pulmonary hypertension of the newborn (PPHN) manifesting with refractory and severe cyanosis is the consequence of high pulmonary vascular resistance (PVR) causing right-to-left shunt of deoxygenated blood through patent ductus arteriosus (PDA) and/or foramen ovale [7]. PPHN is associated with numerous disorders and has rarely been reported as a manifestation of methylmalonic acidemia. We report a newborn with MMA who presented with PPHN.

## Case presentation

A 1-day-old Iranian girl was transferred to our tertiary referral center due to severe cyanosis and refractory metabolic acidosis. She was born in a private hospital in Shiraz, Iran, at a gestational age of 39 + 5 weeks by cesarean section with Apgar scores of 8 and 9 in the 1st and 5th minutes, respectively, from a primigravid, 27-year-old mother with a history of consanguineous marriage. The results of prenatal screening including ultrasound were not significant, and fetal echocardiography was not performed for her. She had a birth weight of 2390 g (< 3rd percentile), length of 50 cm (50th percentile), and head circumference of 34 cm (50th percentile) at birth. She had normal vital signs, including blood pressure of 70/45 mmHg, respiratory rate of 50 breaths per minute, pulse rate of 128 beats per minute, and temperature of 37 °C. She had normal posture, muscle tone, activity, and reflexes such as moro, sucking, rooting, and grasping. Umbilical cord artery blood gas (ABG) revealed pH 7.30, pCO_2_ 47.2 mmHg, pO_2_ 18 mmHg, HCO_3_ 22.7 mmol/l, and base excess (BE) −3.7 mmol/l. She was in a good condition, and breastfeeding started for her immediately after birth. She had normal blood sugar, hemoglobin (15.6 g/dL), and hematocrit (47.7%) on the first postnatal day. Unfortunately, she developed respiratory distress about 10 hours after birth. Hence, she was visited by a pediatrician; she had tachypnea (respiratory rate 85 breaths per minute), cyanosis, retraction (intercostal, subcostal, and supraclavicular), systolic murmur (grade 2 at the apex), and hypotonia on physical examination. Her oxygen saturation was 85%, which increased to 91% with 10-liter oxygen by hood. Therefore, she was transferred to neonatal intensive care unit (NICU) for further evaluation and better management. Chest X-ray was normal, and ABG showed pH 7.25, pCO_2_ 20.4 mmHg, pO_2_ 101.6 mmHg, HCO_3_ 8.8 mmol/l, and BE −18.3 mmol/l. Oxygen saturation decreased to 81% despite oxygen intake after 2 hours, and retraction and tachypnea were intensified. Consequently, the patient was intubated and needed high setup ventilation to maintain acceptable oxygen saturation (more than 90%). Although the patient received appropriate assisted ventilation, her conditions deteriorated and developed severe metabolic acidosis (pH 6.88, pCO_2_ 24 mmHg, pO_2_ 58.6 mmHg, HCO_3_ 4.3 mmol/l, BE −28.8 mmol/l) which was not corrected with hydration and intravenous bicarbonate. Moreover, she developed abnormal movements in her upper extremities. Her blood sugar and calcium were 438 mg/dL and 8.5 mg/dL, respectively, so she was started on phenobarbital. Brain ultrasound was normal. Ammonia, lactate, blood, and urine biochemical tests were requested due to persistent metabolic acidosis. She was transferred to our hospital 36 hours after birth. On arrival, the patient was connected to a ventilator (mode SIMV (Synchronized Intermittent Mandatory Ventilation), PIP (Peak Inspiratory Pressure) 30, PEEP (Positive End-Expiratory Pressure) 7, RR (Respiratory Rate) 60, FiO_2_ (Fraction of inspired oxygen) 100%). Oxygenation index was 29.4. She was hypotensive (blood pressure 42/25 mmHg), and her pulse rate was 145 beats per minute. In our center, the patient had refractory hypoxemia, severe persistent metabolic acidosis, hypotension, and hypotonia. Complete blood count (CBC) showed hemoglobin 12 g/dL with MCV (Mean Corpuscular Volume) 134.1 fl, and normal leukocyte and platelet counts. Results of sepsis workup were negative. Liver function test was normal except mild increase in liver enzymes (SGOT (Serum Glutamic Oxaloacetic Transaminase) 94 and SGPT (Serum Glutamic Pyruvic Transaminase) 71). Her prothrombin time (PT), partial thromboplastin time (PTT), and international normalized ratio (INR) were 39.4 s, 48.7 s, and 2.86, respectively. Serum BUN (Blood Urea Nitrogen), creatinine, and electrolytes were normal. Blood ammonia, lactate, and urine creatinine were 228 μg/dL, 137 mg/dL, and 93 mg/dL, respectively. Echocardiography at the time of admission revealed severe pulmonary hypertension with right-to-left shunt through PDA and foramen ovale, right-side heart failure, and dilated right atrium and ventricle. Bed-side brain sonography showed bilateral ventriculomegaly (ventricular diameter of 23 mm) and intraventricular hemorrhage grade 3 on the 5th day of life. Acylcarnitine profile in the plasma was determined by tandem mass spectroscopy (MS/MS) that showed increased propionylcarnitine (C3), succinyl and methylmalonyl carnitine (C4DC), and low free carnitine (C0) levels. Therefore, the C3/C0 and C4DC/C0 ratios were increased. Amino acid profile in the plasma that was checked by liquid chromatography/tandem mass spectroscopy (LC–MS/MS) showed normal levels of glycine, homocysteine, and other amino acids. Urine organic acid and acylglycine profile was checked by gas chromatography/mass spectrometry (GC/MS) and liquid chromatography (LC–MS/MS). The results showed that lactic acid (824 mmol/molcrt (mmol/ mol creatinine in urine), normal < 90 mmol/molcrt); 3-hydroxybutyric acid (66.1 mmol/molcrt, normal < 5 mmol/molcrt); methylmalonic acid (1052 mmol/molcrt, normal < 18 mmol/molcrt); propionylglycine (8.3 mmol/molcrt, normal < 0.5 mmol/molcrt); 3-hydroxypropionic acid (79.5 mmol/molcrt, normal < 12 mmol/molcrt); methylcitric acid (48.4 mmol/molcrt, normal < 5 mmol/molcrt) were increased. These results, as well as findings of plasma acylcarnitines, were compatible with MMA cobalamin disorders or methylmalonyl-CoA mutase deficiency. The results of these tests were reported on her last day of life.

During the hospital course, the patient received empirical antibiotics with suspicion of sepsis from the first day of admission, bicarbonate to correct metabolic acidosis, milrinone and sildenafil for PPHN, dopamine, dobutamine, and epinephrine due to hypotension, and assisted ventilation due to hypoxemia. Nitric oxide was not available in our center. Metabolic acidosis persisted, so peritoneal dialysis was started for the patient on her 4th day of life. Due to unstable hemodynamic, feeding was not started for the patient. Unfortunately, her oxygen saturation reduced precipitously and did not respond to respiratory and medical support, and she died on her fifth day of life.

## Discussion and conclusion

Our case presented with deterioration of clinical condition in the first postnatal day. Sepsis workup and prenatal history were not in favor of sepsis and pneumonia. Blood sugar, calcium, and other electrolytes were normal. No evidence of asphyxia was detected by Apgar score, umbilical cord ABG, and perinatal history. PDA-dependent congenital heart disease can present with severe refractory cyanosis or shock after spontaneous ductus arteriosus closure. However, the findings of echocardiography in our patient were in favor of the diagnosis of PPHN manifesting with severe and refractory hypoxemia and cyanosis. While the initial suspicion of PPHN arises from the observation of clinical manifestations, such as respiratory or hemodynamic instability in a particular context, the definitive confirmation of PPHN is accomplished through a point-of-care echocardiography that assesses both the structural and functional aspects of the heart [8]. Anatomic heart disease that may mimic PPHN should be evaluated with echocardiography. The predominant direction of shunting from PDA or patent foramen oval should also be determined. Increased right ventricular pressure and tricuspid regurgitation are also signs of PPHN [9]. The associated etiologies of PPHN include lung parenchymal disorders (meconium aspiration syndrome, pneumonia, respiratory distress syndrome), abnormal lung development (pulmonary hypoplasia due to congenital diaphragmatic hernia, oligohydramnios), impaired pulmonary vasodilation at birth (asphyxia, TTN), and 10–20% idiopathic etiology [10]. Our patient had severe refractory acidosis that could not be explained by hypoxemia and sepsis. Elevation of H ^+^ concentration and acidosis produced pulmonary vasoconstriction and increase in PVR and subsequent decrease in pulmonary blood flow, creating extrapulmonary right-to-left shunt and severe cyanosis [11]. The general management of PPHN include hemodynamic stability, avoidance of stress, and empirical antibiotic therapy as difficulty of ruling out sepsis in critically unwell infants. iNO is the first-line therapy to decrease PVR. Sildenafil (phosphodiesterase 5 inhibitor) and milrinone (phosphodiesterase 3 inhibitor) are also used due to pulmonary vasodilation effect [9]. PPHN is rarely reported in patients with inborn error of metabolism (IEM) such as propionic acidemia [12] and mitochondrial diseases [13]. Hence, blood and urine biochemical tests were checked; high concentrations of methylmalonic acid and methylcitrate in the urine, as well as the findings of plasma acylcarnitines of our patient, were compatible with MMA. MMA is a rare autosomal recessive disorder with an incidence rate of 1:50,000 [3]. Although there was no family history of MMA in the patient, she was the product of a consanguineous marriage. In MMA, deficiency of MCM enzyme or AdoCbl coenzyme of vitamin B12 leads to accumulation of methylmalonic acid in the body. Diagnosis of MMA is made through investigation of metabolites with tandem mass spectrometry, organic acid analysis with gas chromatography, enzymatic studies with fibroblast cell culture, and finally mutation analysis [3]. Subtypes of MMA are diagnosed by enzyme assay and/or molecular genetic techniques [3]. We did not check these tests in our patient due to limited time and high cost. However, very high concentrations of methylmalonic acid (> 1000 mmol/molcrt) in the urine with normal homocysteine were in favor of isolated MMA (mut-, mut0, cblA, and cblB) subtypes. The patient had mild megaloblastic anemia, which raised the possibility of cobalamine defect.

Agarwal *et al.* reported the first case of MMA presenting with PPHN in 2014 [14]. They reported the case of a term neonate with meconium-stained amniotic fluid who was healthy at birth but developed severe respiratory distress after a short time. PPHN and MMA were confirmed on echocardiography and neonatal screening consequently. According to chest X-ray and autopsy findings, meconium aspiration syndrome was not the cause of PPHN, and evidence of hypertrophy and intimal hyperplasia was seen in pulmonary arterioles. Moreover, there are reports of pulmonary artery hypertension (PAH) in cases of MMA that did not present during the neonatal period. Chioukh *et al.* [15] reported the case of a 16-month-old male infant admitted with PAH and biochemical-test-confirmed MMA. The patient died despite treatment. Kido *et al.* [16] reported the case of a woman known to have had MMA at the age of 3 months, which was complicated with PAH at a 36 years of age. Their patient was treated successfully. Most case reports of MMA with cardiovascular diseases have combined MMA and homocystinuria such as eight cases reported by Liu *et al.* [6]. The mechanism has not been elucidated, but it may be due to vasculopathy and thrombosis [17]. In a cohort study of 301 Chinese patients with isolated MMA, six patients had cardiomyopathy and two of them developed PAH [2]. Other case reports with isolated MMA and cardiovascular manifestations are presented in Table 1. Although our patient had normal brain sonography on the first day of life, she developed IVH (Intra-Ventricular Hemorrhage) grade 3 on her last day of her life. Cerebral hemorrhage has been reported as a rare manifestation that could be due to correction of acidosis [18].

**Table 1:** Review of the articles (cases reported with isolated MMA and cardiac manifestations)

Authors and references	Age at MMA diagnosis	Age at cardiac disease diagnosis	Sex	Manifestations of MMA	Cardiovascular manifestations	Outcome
Prada *et al.* [5]	4 months	22 years	F	Developmental delay Chronic tubulointerstitial nephropathy ESRD	Pericardial effusion LV hypertrophy Cardiomegaly EF: 18%	Died
Prada *et al.* [5]	2 days	7 months	M	Tachypnea Hyperglycemia Vomiting	Cardiomyopathy Aortic stenosis EF: 20–42%	Died
Prada *et al.* [5]	Neonate	4 years	F	Kusmal respiration Metabolic acidosis Hyperammonemia Renal tubular acidosis	Supraventricular tachycardia Dilated cardiomyopathy EF: 20–25%	Died
Chioukh *et al.* [15]	16 months	16 months	M	Fever Cough Tachypnea Cyanosis	Mild pericardial effusion PAH Enlarged right ventricle	Died
Agarwal *et al.* [14]	Neonate	Neonate	F	Respiratory distress Acidosis	PPHN	Died
Kido *et al.* [16]	3 months	35 years	F	Vomiting Lethargy Tachypnea Renal failure	PAH	Survived

EF, ejection fraction; ESRD, end-stage renal disease; F, female; LV, left ventricle; M, male; PAH, pulmonary arterial hypertension; PPHN, persistent pulmonary hypertension of the newborn

Our limitations in diagnosis and treatment include lack of prenatal diagnosis and delay in test results such as ammonia and biochemical tests. Furthermore, due to the short lifespan of the patient, some tests were not repeated. Regarding treatment, inhaled nitric oxide, the first line of PPHN treatment, was not available in our country, so other drugs were used to control PPHN. The patient’s family received genetic counseling. However, they did not undergo genetic tests due to economic problems.

Severe IEM may cause irreversible damage with adverse lifelong morbidity, and early diagnosis may help to prevent such complications. Furthermore, diagnosis of these disorders helps prenatal intervention for subsequent pregnancies. Knowledge of the association between PPHN and IEM can help to consider underlying IEM and its management in some cases of idiopathic PPHN.

## Data Availability

Materials and data provided in this case study are available from the corresponding author upon reasonable request.

## Abbreviations

ABG: Arterial blood gas
IEM: Inborn errors of metabolism
MMA: Methylmalonic acidemia
PAH: Pulmonary arterial hypertension
PDA: Patent ductus arteriosus
PPHN: Persistent pulmonary hypertension of the newborn
PVR: Pulmonary vascular resistance
TTN: Transient tachypnea of neonate
MCM: Methylmalonyl COA mutase
AdoCbl: Adenosylcobalamin
NICU: Neonatal intensive care unit
CBC: Complete blood count
PT: Prothrombin time
PTT: Partial thromboplastin time
INR: International normalized ratio
